# Perspectives of Microstructure Refinement of Aluminum and Its Alloys by the Reciprocating Extrusion (Cyclic Extrusion Compression—CEC)

**DOI:** 10.3390/ma15114006

**Published:** 2022-06-04

**Authors:** Maria Richert, Rafał Hubicki, Piotr Łebkowski

**Affiliations:** 1 Akademia Górniczo-Hutnicza w Krakowie, ul. Gramatyka 10, 30-067 Krakow, Poland; plebkowski@agh.edu.pl; 2 Grupa Kęty S.A., ul. Kościuszki 111, 32-650 Kety, Poland; rhubicki@grupakety.com

**Keywords:** structure, nanomaterials, shear bands, steady state flow, aluminum, aluminum alloys, severe plastic deformation (SPD), reciprocating extrusion (cyclic extrusion compression—CEC)

## Abstract

This paper presents a study on the perspectives of structure refinement of aluminum and its alloys by reciprocating extrusion (cyclic extrusion compression—CEC). The study included Al99.5 and Al99.992 aluminum and AlMg5 and AlCu4Zr alloy. Aluminum and alloys were deformed by reciprocating extrusion (CEC) in the strain range ϕ = 0.42 (1 CEC cycle) to ϕ = 59.8 (67 CEC cycles). After deformation, the structure of the specimens was investigated by optical microscopy (OM) and transmission electron microscopy (TEM), which revealed that the primary mechanism of hardening, over the range of applied strains, was the result of the propagation of shear bands throughout the specimens. The intersection of shear bands was found to divide the volume of the specimens into nano and microvolumes with dimensions limited by the width of the microbands. Due to structure renewal processes such as polygonization and dynamic geometric recrystallization, the formed micro and nano volumes were transformed into nano and micrograins with large misorientation angles. In terms of the occurrence of grain microstructure, a sustained uniform level of hardening was found, which was defined as steady-state flow. The research has shown that the steady state of flow is a result of the competitive interaction between the processes of hardening and structure renewal. The higher the metal purity, the higher the intensity of the structure renewal processes was. The formation of new grains and their growth under dynamic and post-dynamic recrystallization was observed in Al99.992 aluminum, in which high purity of the metal and high strain accumulation caused the growth of new grains at room temperature.

## 1. Introduction

Severe plastic deformation involves exerting arbitrarily large plastic strain without changing the shape of the specimen being deformed. This type of deformation is provided by two methods: reciprocating extrusion (cyclic extrusion compression—CEC) [1,2,3] and equal channel angular extrusion [4,5,6,7]. Both methods are characterized by the presence of comprehensive compressive stresses during deformation.

In the method that applies large deformation in other states of stress, named constrained groove pressing (CGP), material is subjected to the repetitive shear deformation under the plane strain deformation condition by utilizing alternate pressing with the asymmetrically grooved die and flat die constrained tightly by the cylinder wall [8,9].

It should be emphasized that the realization of deformation by CEC and ECAP methods requires the use of special presses. This is particularly true for the CEC method, in which versatile compressive stresses are exerted and maintained during deformation [10,11].

Many studies have been conducted to determine the changes in structure and properties of materials subjected to severe plastic deformation. It has been found that the material hardens during the first few cycles of deformation and then transitions to a steady-state flow that is characterized by almost uniform property levels [12,13]. In the steady state, the structure reflects the property level and remains stable in terms of grain size and dislocation density. It is evident that steady-state flow is the result of a balance between the softening (recovery and recrystallization) and hardening processes. The type of metal or alloy being deformed also affects the level of steady-state flow. The higher the stacking fault energy, the faster the steady-state flow is achieved, and the lower the level of hardening in this state [14,15].

The concept of mass production of volumetric nanometric materials by plastic deformation of metals or alloys by SPD methods has encountered limitations related to the sample size. Deformation by CEC and ECAP methods of large volumes of metals requires very high forces. The second obstacle is the natural structural changes and the resulting property level, which is the result of competing deformation mechanisms and structure renewal mechanisms, leading to property stabilization and no further hardening.

As a result of the accumulation of extremely large strains, there is also a strong decrease in the recrystallization temperature which decreases the threshold for the possibility of maintaining the deformation structure and high values of hardening, especially in very pure metals. In extreme cases, when conditions are created for the development of softening mechanisms, new grains, free of dislocations, are formed, and even growth of the new grains is observed [16,17,18]. At the same time, with the increase in strain, the temperature of the deformed material increases, which further promotes the development of structure renewal processes and softening of metals [19]. The resulting nanometric structure is not stable, especially in metals and alloys characterized by high stacking fault energy [20].

These phenomena, in extreme cases, lead to the impossibility of grain refinement of some materials. Therefore, it has been postulated that only certain alloys, in which the development of recovery and recrystallization processes is hindered, may potentially offer the possibility of producing nanometric structure in the deformed material [21]. Examples include Mg-AZ31 alloys [21], austenitic steels [22], steel, Cu, Ni [23,24], Al7.5%Zn-2.7%Mg-2.3%Cu-0.15%Zr alloy [25], (Ni_1.5_FeCoCr_0.5_)_87.5_Al_7.5_Ti_5.0_ [26], Cu-Fe-P, Cu-Ni-Si, Cu-Cr-Zr [27]. Studies have shown that the formation of nanostructure is associated with an increase in the level of hardening (Ni_1.5_FeCoCr_0.5_)_87.5_Al_7.5_Ti_5.0_. It is characteristic to reach a steady-state flow in the range of very high deformation, after an initial strong increase in hardening [28,29]. The deformation and hardening mechanisms in the extremely high strain range leading to the formation of nanometric structures are slip, twinning, and grain boundary sliding [30].

The study of Dobatkin et al. [31] demonstrated the capability of producing a nanostructure in steel by torsion under high pressure (HPT) and equal channel angular (ECA) pressing, which makes it possible to obtain high strength and sufficient ductility. The characteristic feature of the microstructure was elongated grains shaped in bands. Obtaining nanostructures using SPD processes was also reported in the study of Zhu and Langdon [32]. Nanostructures were also obtained in aluminum alloys deformed by CEC method [33]. In the work of Besterci et al., after 10 cycles of ECAP, structure refinement was obtained in copper to 100–300 nm [34]. In ECAP-deformed magnesium alloys, the grain refinement occurred by recrystallization initiated at grain boundaries and in shear bands [35]. The new grains formed were nanometric in size.

The paper presents the results of research on the possibility of structure refinement in Al99.992 and Al99.5 aluminum and its alloys AlMg5 and AlCu4Zr, deformed by reciprocating extrusion (cyclic extrusion compression—CEC). In the light of the obtained results and literature data, the possibility of structure refinement in aluminum and its alloys has been analyzed, as well as the potential for a practical use of the applied deformation method in practice.

## 2. Materials and Methods

**Figure 1:**
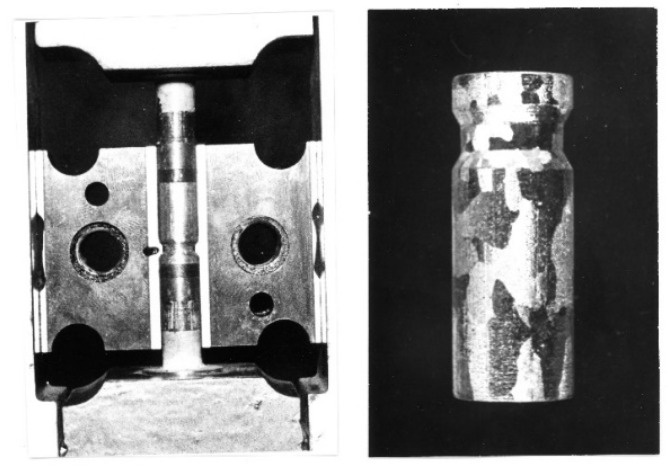
Initial specimen placed in a split ECAC extrusion die, initial specimen shape.

The tests were carried out on high-purity aluminum Al99.992, technical aluminum Al99.5 and AlMg5, and AlCu4Zr alloys. The specimens, with initial shape matching the die shape (Figure 1), were deformed by reciprocating extrusion (cyclic extrusion compression—CEC) using the new CEC press.

The die, along with the sample, was placed in a container on a CEC extrusion press. In order to ensure that the versatile hydrostatic stresses were applied, the specimen was closely fitted to the shape of the die, which was enclosed in a housing and compressed before the deformation process to ensure that the containers were completely filled with the material to be deformed. With this arrangement, very large plastic strains could be exerted without loss of specimen integrity.

**Figure 2:**
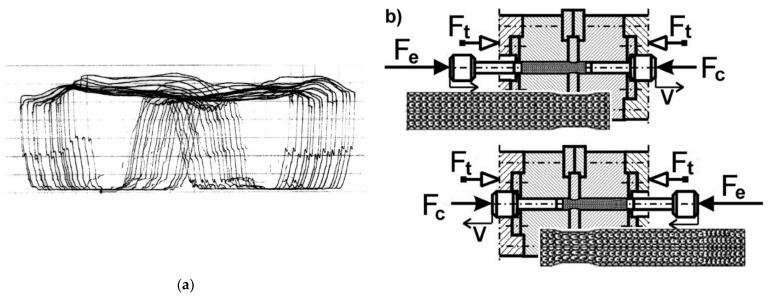
Force diagram of the CEC process (**a**), each wing of the “butterfly diagram” corresponds to the displacement of the specimen from one container of the CEC matrix to another, e.g., from left to right (**b**).

During deformation, the force exerted on the specimen was measured and recorded during the CEC process (Figure 2a). Each wing of the “butterfly” diagram (Figure 2a) corresponds to one extrusion cycle of the specimen. For example: when a specimen is extruded with the force F_e_ from the left to the right container (Figure 2b), the force F_t_ is the force holding the die in a state of versatile compressive stress, and the force F_c_ is the counter force to the force F_e_ holding the specimen in the die and ensuring that the die space is tightly filled with specimen material. The diameter of the containers d_o_ = 10 mm and the diameter of the die orifice d_m_ = 9 mm, d_m_ = 8.5 mm, and d_m_ = 8 mm were used.

The specimens were 10 mm in diameter and approximately 50 mm long (Figure 1).

Specimens were deformed over the range of true plastic strain (denoted by φ) φ = 0.42 (1 CEC cycle) to φ = 59.8 (67 CEC cycles).

The strain was calculated according to the formula [2,10]:

φ = 4 n ln d_o_/d_m_ (1)

where n—number of cycles of CEC process

d_o_—initial diameter of the sample

d_m_—diameter of the CEC die orifice

In the work, aluminum Al99.992 and Al99.95 samples were deformed up to the accumulated strain (denoted by φ) φ = 59.8. The channel diameter was d_m_ = 8.0 mm, which corresponds to a strain φ = 4 ln (d_0_/d_m_) = 0.89 exerted in a single CEC cycle. Alloy AlMg5 was deformed up to the accumulated strain φ = 16. The channel diameter was d_m_ = 8.5 mm, which corresponds to a strain increment φ = 4 ln(d_0_/d_m_) = 0.65 exerted in a single CEC cycle. Alloy AlCu4Zr was deformed up to the accumulated strain φ = 14. The channel diameter was d_m_ = 9.0 mm, which corresponds to a strain increment φ = 4 ln (d_0_/d_m_) = 0.42 exerted in a single CEC cycle.

After deformation, the samples were tested for yield stress in the compression test and their microstructure was examined by means of both optical and transmission electron microscopy. The investigations were performed on the longitudinal sections of specimens. The microstructure was observed in the middle part of longitudinal section of samples. The structure of samples was examined with an Olympus GX51 optical microscope (Olympus, Tokyo, Japan).

Samples for microstructure examinations by optical microscopy (OP) were mechanically ground and polished with diamond paste and a colloidal suspension of SiO_2_. The microstructure was revealed by the technique of etching with Barker reagent. The composition of the Barker reagent was as follows: 1.8 cm^3^ HBF_4_ + 100 mL H_2_O.

The microstructure was investigated on thin foils using transmission electron microscope JEOL2010 ARP. The special software KILIN [36] was used to determine misorientation on the base of the Kikuchi diffraction patterns.

The thin foils were cut out from longitudinal sections of the samples and prepared applying the standard technique of electrolytic polishing using the Struers apparatus. Additionally, the statistical width of the microbands and micro and nanograins observed in the microstructure was calculated using the mean chord method. The misorientation of selected microstructural elements was determined using proprietary KILIN software.

Vickers microhardness (HV) was measured on mirror-shine polished sample surfaces using a microhardness tester PMT3. A load of 100 g was applied in the hardness measurements.

The yield point of aluminum and AlMg5 alloy was tested in a compression test. Cylindrical specimens 6 mm high were cut from CEC deformed specimens and compressed on an Instron testing machine at a strain rate of 10^−2^ s^−1^. Graphite lubricant was applied to the face surfaces of the specimens.

The size of grains/nanograins has been determined by the method mean chord measuring.

## 3. Results

**Figure 3:**
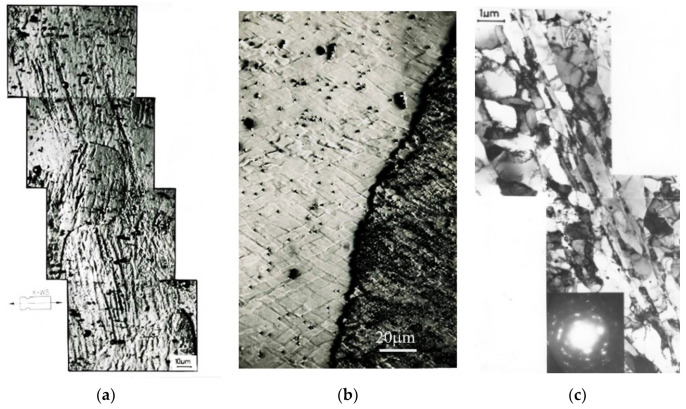
Microstructure of Al99.5 after CEC deformation, φ = 4.5 (5 CEC cycles). Extrusion direction parallel to the long edge of the structure photo (**b**,**c**). (**a**) Shear bands running through several grains in Al99.5 (OM). (**b**) Network of crossing shear bands within grains in Al99.5 (OM). (**c**) In-grain shear bands in Al99.5 (TEM).

**Figure 4:**
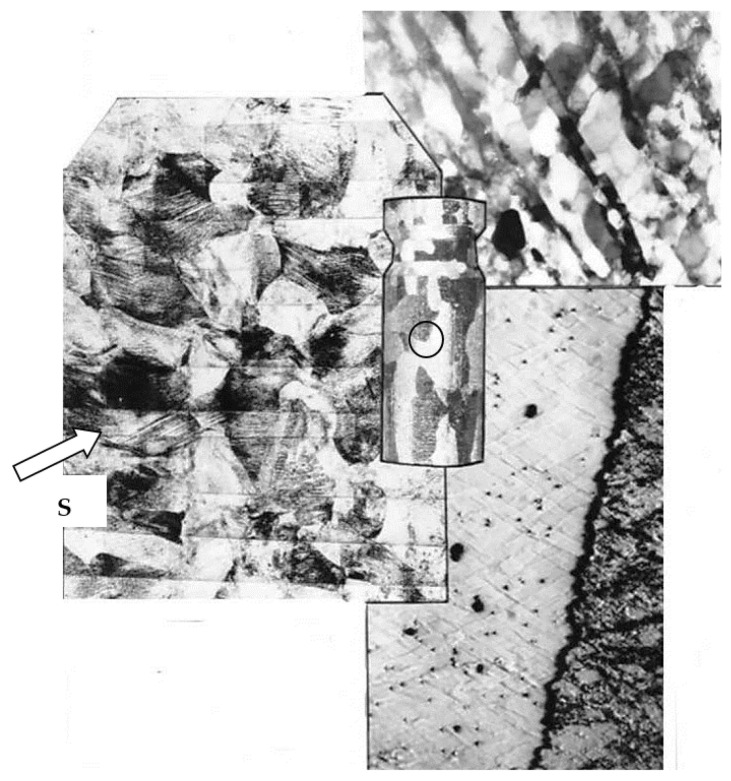
Characteristic band structure of Al99.5 after deformation by CEC method up to 5 deformation cycles, φ = 4.5, S—Shear Band. Arrowhead indicates the propagation direction of the shear band. The place where the structure was examined is marked with a circle.

Figure 3 shows a typical microstructure of Al99.5 aluminum after CEC deformation (φ = 4.45; 5 cycles of CEC deformation) with characteristic shear bands that run rectilinearly through many grains (Figure 3a). Shear bands were observed in all deformed samples. The results of the performed tests, as well as the literature data, prove that this is the basic deformation mechanism in the range of extremely high SPD deformations. Noteworthy is the long-range course of shear bands through the specimen. This course of bands can be observed in the macroscopic structure map made with the optical microscope. The longitudinal section map is shown in Figure 4, where the broad macroscopic shear band is indicated by an arrow and the letters SB. The bands are transposed through the whole section of the sample in two opposite directions. They form bundles of several, a dozen, or more individual bands.

Long-range slip propagation in the shear bands, mutually intersecting, led to the formation of characteristic rhomboid-shaped micro-areas contained between the crossing bands, which were observed inside the aluminum grains (Figure 3b and Figure 4).

**Figure 5:**
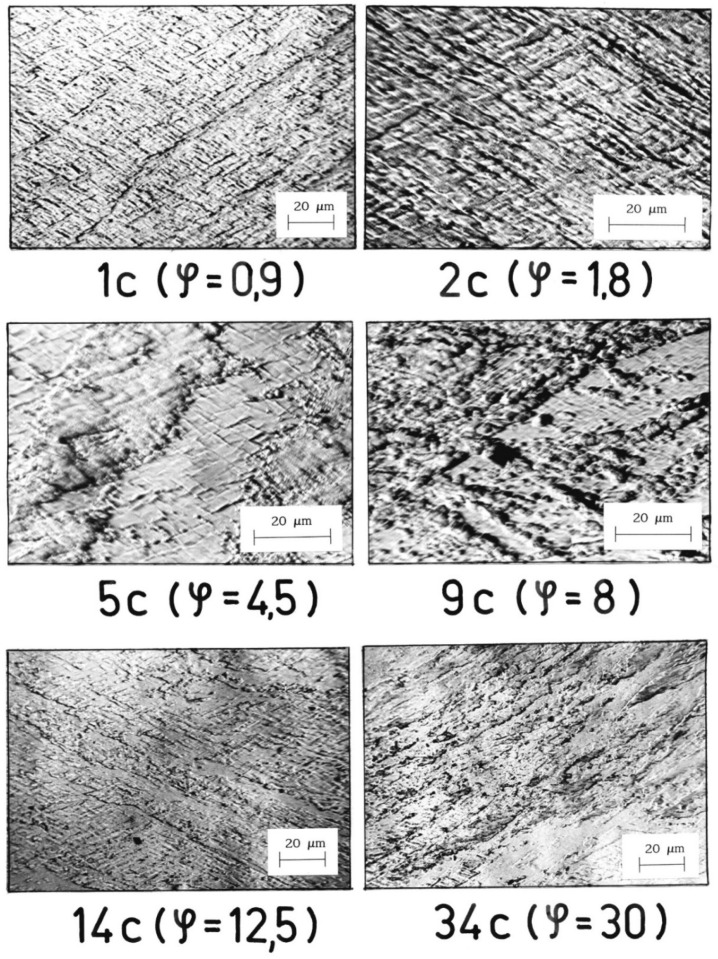
Crossing bands in Al99.5 forming characteristic rhomboid-shaped microvolumes between the bands. The direction of extrusion is consistent with the short edge of the photo.

The band structure was formed in Al99.5 aluminum from the first cycle of deformation performed by CEC method (Figure 5). Additionally, from the first cycle of deformation, areas of characteristic structure were observed, resulting from the mechanism of crossing bands, examples for which successive specimens with increasing strain are shown in Figure 5. With increasing strain, the volume of specimens occupied by this type of structure increased.

**Figure 6:**
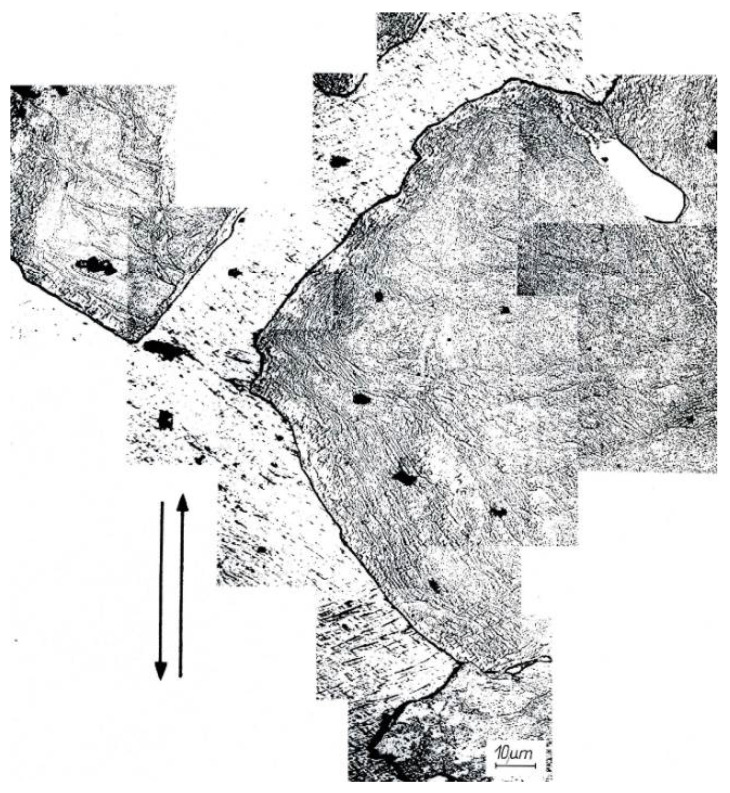
Structure of Al99.992 after 2 CEC cycles—φ = 1.8. The arrows indicate extrusion direction in CEC.

**Figure 7:**
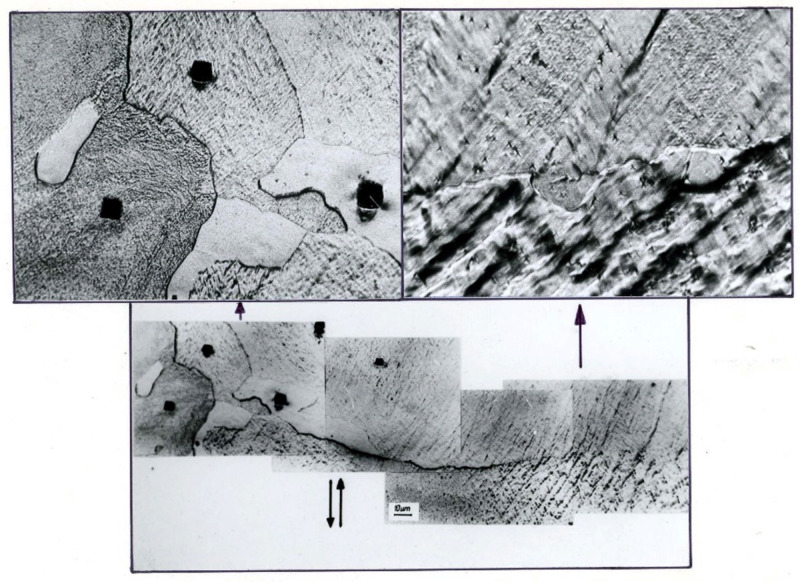
Structure of Al99.992 aluminum after 2 CEC cycles—φ = 1.8, arrows indicate larger sections of the structure. The double arrows indicate the direction of extrusion.

A banded structure and recrystallized areas were found in Al99.992 aluminum after the second deformation cycle (φ = 1.8) (Figure 6). There were very strongly defected areas in the specimen with a clearly visible band structure. New recrystallized grains were observed inside the defected grains. Detailed examination revealed that the nucleation of new grains occurs at the intersections of shear bands with grain boundaries (Figure 7). On the enlarged fragment of the structure, new grains located inside the bands, grouped at the old grain boundary, are noticeable. The microhardness indentations show that the new grains have the lowest microhardness. The highest microhardness (smallest penetrator indentations) was found in the most defected areas.

**Figure 8:**
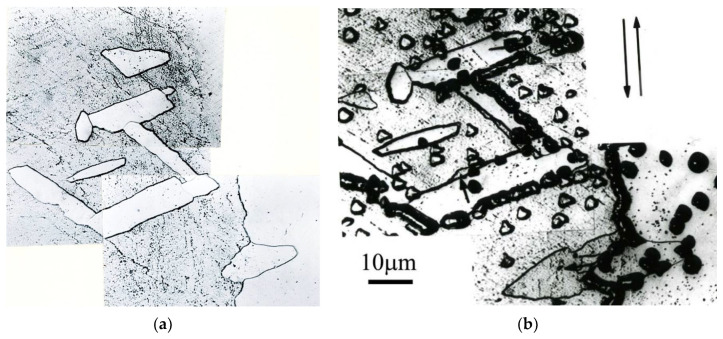
Group of new grains in Al99.992 which were formed by dynamic recrystallization during CEC deformation, (**a**) new grains oriented according to the shear bands, (**b**) growth of new grains after holding the sample for 12 days at room temperature, the direction of boundary displacement is marked by arrows. The arrows indicate the direction of extrusion in CEC.

Al99.992 aluminum samples after CEC deformation were held at room temperature for 12 days and then re-etched and observed. Figure 8a shows the structure immediately after deformation, and Figure 8b shows the same section of the structure after 12 days of holding at room temperature. The arrows indicate where the boundaries of new grains have moved, probably formed by dynamic or post-dynamic recrystallization. The high purity of aluminum and the very high strain φ = 1.8 activated the nucleation of new grains and, after deformation, allowed them to grow deep into the heavily defected structure. Room temperature was sufficient for the growth of the newly formed grains.

**Figure 9:**
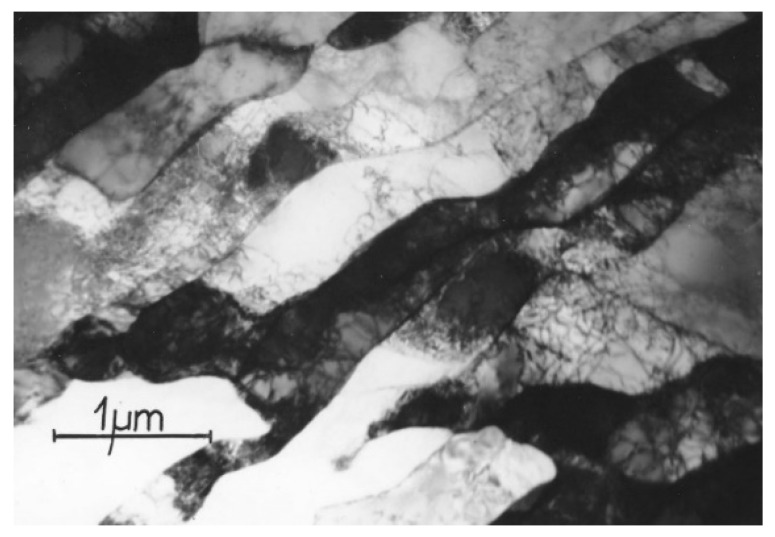
Faulting at the microband boundaries in Al99.5 aluminum after deformation of φ = 1.8, 2 CEC cycles. The direction of extrusion is consistent with the short edge of the photo.

The transformation under the influence of changes caused by the propagation of shear bands produced a qualitatively new microstructure. Investigations by transmission electron microscopy showed that in Al99.5 aluminum the shear bands are composed of elongated subgrains (Figure 9). The width of these subgrains was in the range of 200–900 nm. A characteristic deflection of the microband boundaries (faults at the subgrain boundaries) was observed, which resulted from the shear mechanism. The bands ran linearly in two opposite directions and crossed each other. The shear mechanism caused displacement of the microband walls and division of the microbands into microvolumes bounded by the microband walls.

**Figure 10:**
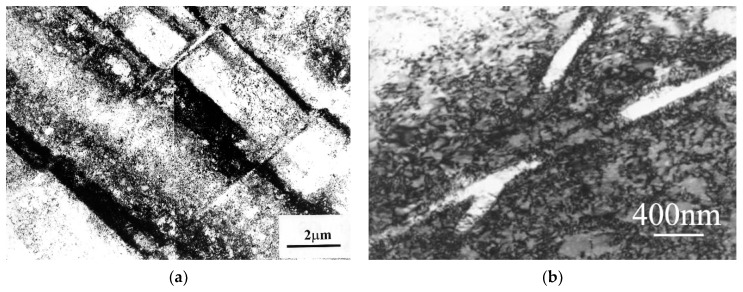
Crossing microbands in AlMg5 alloy, (**a**) microstructure divided by crossing microbands, (**b**) microbands with nanometer width—about 100 nm. The direction of extrusion is consistent with the short edge of the photo.

In AlMg5 alloy, the microbands were much narrower than those found in Al99.5 aluminum. They were about 100–200 nm wide. They ran against a background of a strongly defected dislocation structure (Figure 10). The microbands at the change of the foil inclination showed a low dislocation density compared to the surrounding material. Figure 10a shows a typical microstructure of AlMg5 alloy with numerous narrow microbands that divided the microstructure into rhomboid-shaped regions. Similar to Al99.5 aluminum, characteristic faults at the boundaries of the microbands were also observed in the AlMg5 alloy. AlMg5 alloy exhibited a significant dislocation density. On the background of dislocation densities there were microbands of about 100 nm width (Figure 10b).

**Figure 11:**
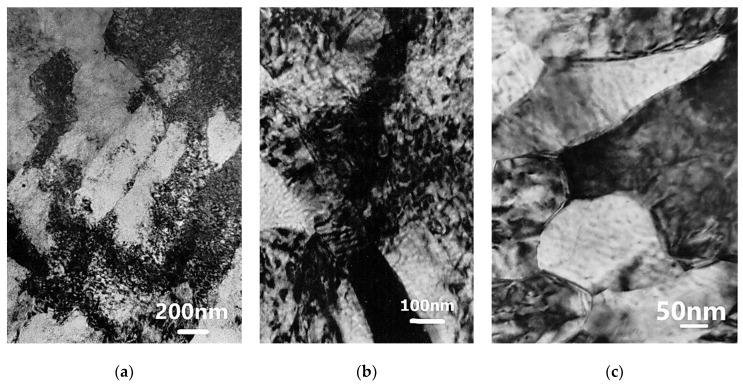
Microstructure of AlCu4Zr alloy with bands about 100–200 nm wide, (**a**) after deformation of φ = 4.64 (11 CEC cycles), (**b**) crossing microbands after deformation of φ = 4.64 (11 CEC cycles), (**c**) after deformation of φ = 14 (33 CEC cycles). The direction of extrusion is consistent with the short edge of the photo.

The microstructure of the AlCu4Zr alloy contained microbands composed of elongated subgrains and areas of mutually intersecting microbands (Figure 11). The microbands in the AlCu4Zr alloy had widths ranging from tens to hundreds of nanometers. After 33 cycles of deformation (φ = 14), significant areas of microstructure consisting of grains with dimensions of about 100–150 nanometers were observed in the AlCu4Zr alloy.

**Figure 12:**
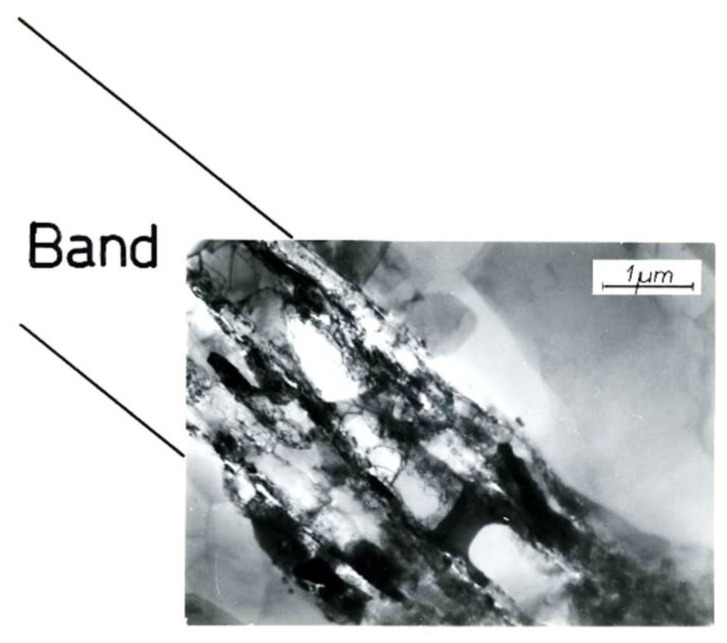
The banded microstructure in Al99.992 after 2 cycles of CEC (φ = 1.8). The direction of extrusion is consistent with the short edge of the photo.

In Al99.992, a banded microstructure consisting of elongated subgrains of varying width from about several hundred nanometers to one micrometer or more was found (Figure 12). The microbands exhibited a diverse internal microstructure containing highly defected areas and micro-areas completely cleared of dislocations, which should be considered as potential nuclei of new grains.

**Figure 13:**
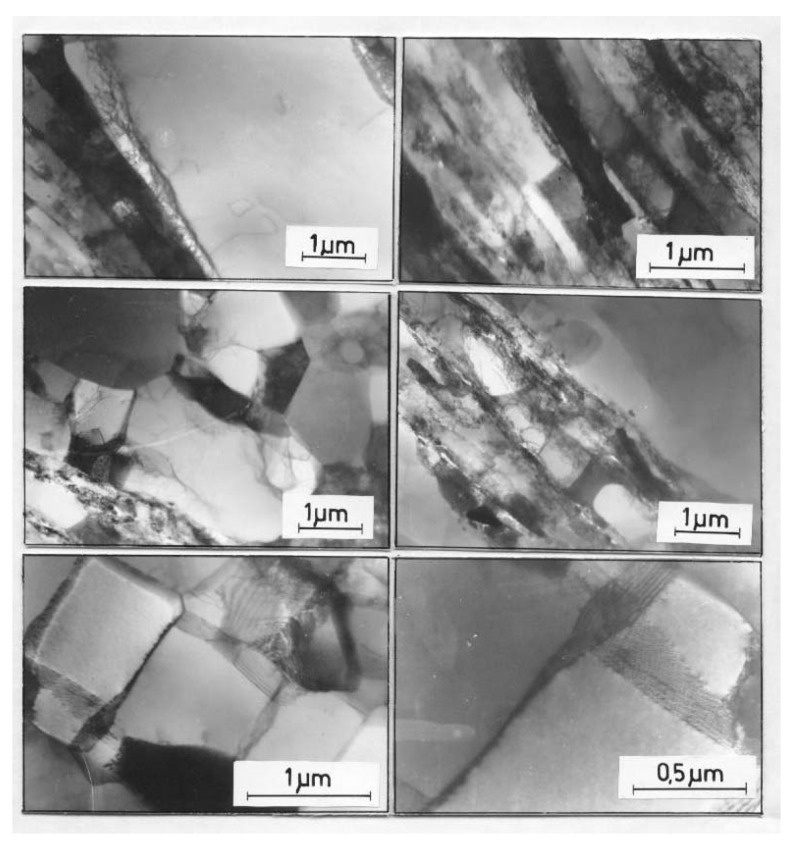
Microstructure of Al99.992 after 2 cycles of CEC (φ = 1.8). The direction of extrusion is consistent with the short edge of the photo.

The dislocation-free subgrains/grains observed inside the microbands were oriented according to the direction of the microband course (Figure 13). They were usually parallelograms in shape, from several to several dozen micrometers wide and tens of micrometers long. They were located between the microbands or within the microbands. Example structures presented in Figure 13 show areas of dislocation-free subgrains/grains with equiaxed shapes adjacent to strongly defected microbands.

The observed microstructure image shows the effects of dynamic recrystallization occurring during deformation. High purity of the metal and high accumulated energy led to the development of structure renewal processes [37,38]. Microbands became the site of nucleation and growth of new grains in Al99.992 aluminum (Figure 8). The recrystallized grains reflected the alignment geometry of the crossing shear bands. They assumed shapes and growth direction consistent with the crossing bands. The growth of new grains followed the direction of the shear bands. This indicates significant mobility of the new boundaries formed perpendicular to the walls of the microbands.

**Figure 14:**
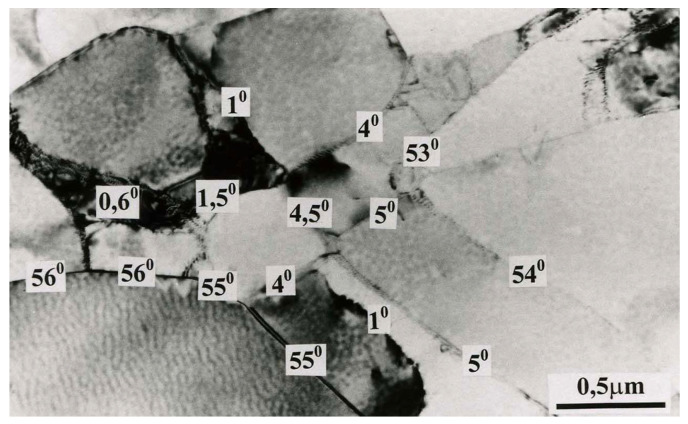
The grain structure in Al99.5 aluminum after deformation of φ = 22.3 (25 cycles of CEC). The direction of extrusion is consistent with the short edge of the photo.

**Figure 15:**
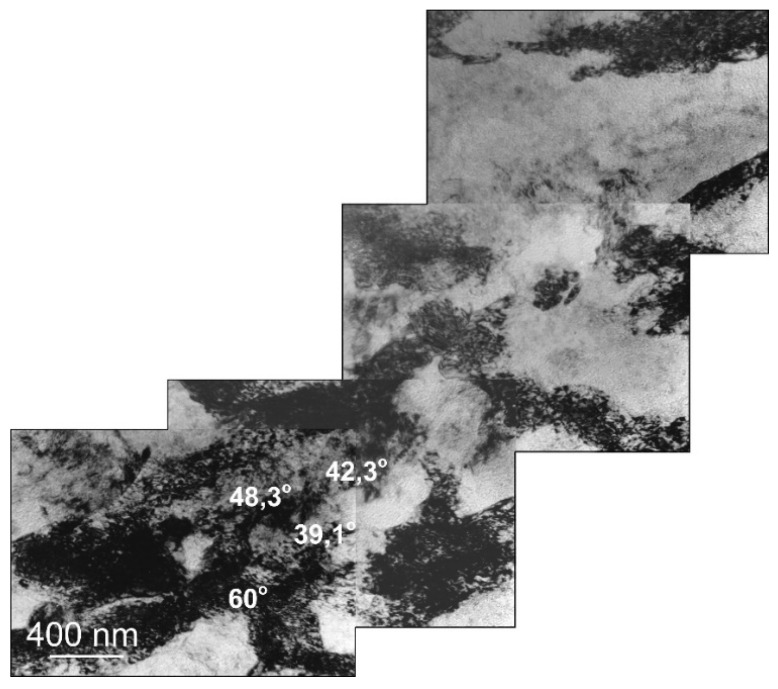
The microstructure of AlMgSi alloy after deformation of φ = 16, area of nanograins with high misorientation. The direction of extrusion is consistent with the short edge of the single photo.

**Figure 16:**
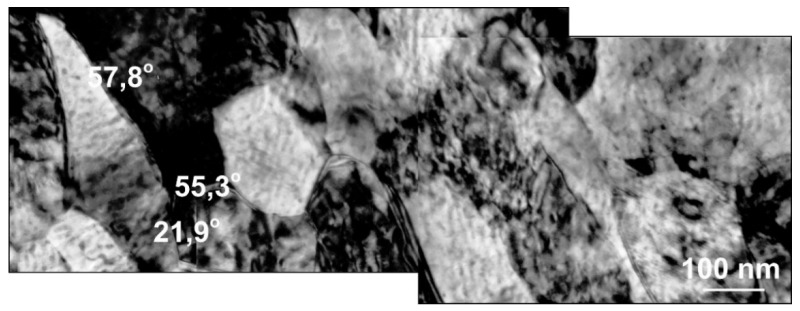
The microstructure of AlCu4Zr alloy after deformation of φ = 14, the area of nanograins with high misorientation. The direction of extrusion is consistent with the short edge of the photo.

Misorientation studies were performed in the band structure regions in Al99.5 aluminum, AlMg5 alloy, and AlCu4Zr alloy using transmission electron microscopy. The misorientation was determined by Kikuchi diffraction using a specially developed program. The values of misorientation angles were found to vary from low to high disorientation angles. Example results are shown in Figure 14 for Al99.5 aluminum, in Figure 15 for AlMg5 alloy, and in Figure 16 for AlCu4Zr alloy.

**Figure 17:**
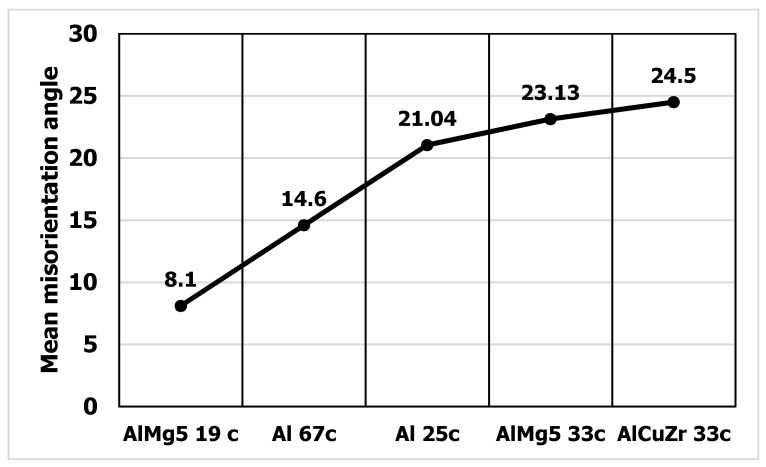
Mean misorientation angle in aluminum and aluminum alloys after CEC deformation.

A summary statistical study of the misorientation angles was averaged and illustrated in a graph (Figure 17). Large misorientation angles with an average value of about 21° occurred in Al99.5 aluminum after 25 cycles of deformation, while after 67 cycles of deformation the average misorientation angle in Al99.5 aluminum was 14.6°.

For the AlMg5 alloy, the misorientation angle increased with increasing strain. After deformation φ = 8 (19 CEC cycles), the misorientation was 8.1°, while after 33 cycles, φ = 13.9 it was 23.3°. The highest values of misorientation angles occurred in the AlCu4Zr alloy, which averaged 24.5° after deformation of φ = 14 (33 CEC cycles).

The data obtained show that with increasing strain for AlMg5 and AlCu4Zr alloys, the misorientation angles increased. For Al99.5 aluminum, larger misorientation angles occurred at lower strain.

**Figure 18:**
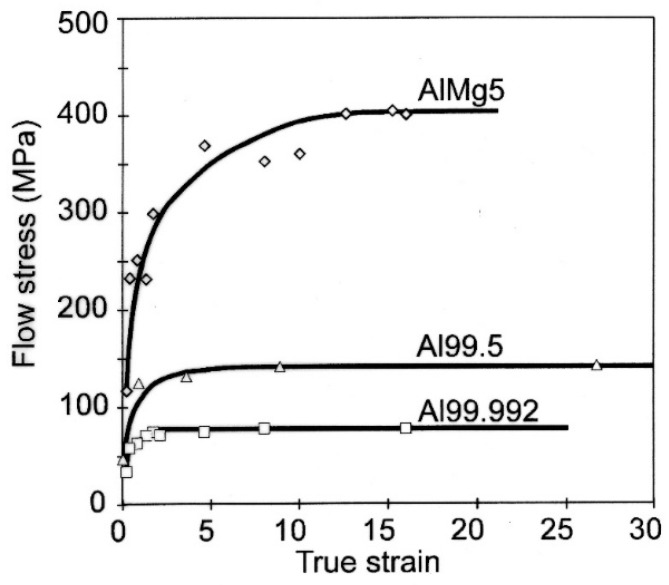
Yield strength of aluminum and AlMg5 alloy after CEC deformation.

The course of changes in the yield strength of the specimens determined in the compression test is shown in Figure 18. After an initial strong increase in hardening, depending on the type of strained material, a steady-state flow condition was found, which was characterized by an almost uniform level of flow stress. The steady-state property level depended on the type of metal being deformed. The lowest steady-state hardening was achieved in Al99.992 aluminum (about σ = 75 MPa), higher hardening was achieved in Al99.5 aluminum (σ = 140 MPa), and the highest hardening was exhibited by AlMg5 alloy (σ = 410 MPa)). The initial increase in hardening, until reaching the steady state, lasted for Al99.992 aluminum until about φ = 1.8 (2 CEC cycles). For Al99.5 aluminum, steady-state flow occurred after 5 CEC cycles (φ = 4.5), while for the AlMg5 alloy, steady-state flow was achieved after deformation of φ = 16 (33 CEC cycles).

**Figure 19:**
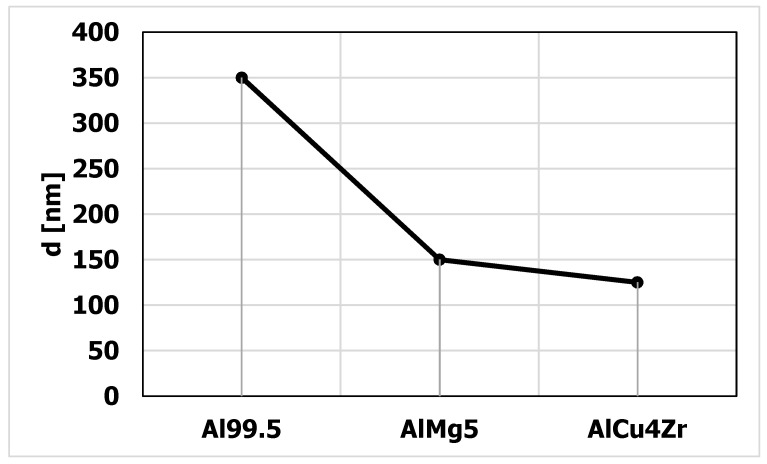
Grain/nanograin size after CEC deformation, in the range of steady-state flow. The size of grains/nanograins has been determined by the method mean chord measuring.

In terms of steady state flow, the microstructure was characterized by the presence of nano and micrograins. The average size of the grains formed in the steady state flow is shown in Figure 19. The smallest average grain size was found in the AlCu4Zr alloy (d = 125 nm), in the AlMg5 alloy the grain size was d = 150 nm, and in the Al99.5 aluminum it was d = 350 nm.

The results indicate that the primary deformation mechanism in the reciprocating extrusion CEC method, as in other SPD methods, is shear slip in the shear bands. Shear bands develop from the onset of deformation and successively fill the volume of the specimens, causing a complete remodeling of the microstructure. Once steady-state flow is reached, the hardening process competes with structure renewal processes such as polygonization and geometric dynamic recrystallization leading to the formation of a granular structure [39,40]. Depending on the type of material, purity, presence of precipitates, and impurities, the resulting grain structure is close to 100 nm in size or exhibits micrometric sizes.

## 4. Discussion

Richert presented the mechanism of shear band formation in the CEC process and determined the actual strain and stress state occurring in the deformation zone [39]. The structure studies showed that the shear band propagation mechanism is the main deformation mechanism in metals and alloys deformed by CEC.

In other works on exerting severe plastic strain by SPD methods, a similar phenomenon of dominance of shear mechanism in structure formation in deformed metals and alloys was found. A strongly extended band structure was observed by electron microscopy in AA3104 aluminum severely deformed by ECAP method [40]. In the work of Zrnik et al. it has been shown that the banded elongated subgrain structure is present due to dominant shear strain [41]. They also suggested that increased processing temperature effect is flow softening of the material, which may lead to plastic flow localization and to fracture. Segal indicated that computed and experimental results confirmed that the average grain rotation and refinement were much more intensive for simple shear during ECAE than for pure shear during rolling [42].

In the work of Jia et al., 2017, Al-5 wt.% Cu alloy containing a small fraction of Al deformed by ECAP method was studied [43]. The study showed that with the increase in strain, the grain refinement occurs and the hardness of the alloy increases. The microstructure analysis revealed that during ECAP, the grain refinement occurs predominantly due to the subdivision of original coarse grains by forming shear bands and by continuous dynamic recrystallization (CDRX).

Prangnell et al. found that in high stacking fault energy metals, the refinement of the structure in SPD processes such as ECAP occurs as a result of orientation splitting, micro and macro shear bands formation, and as a result of geometric requirements resulting from an increase in the angle of boundary misorientation as strain increases [44].

In the ECAP deformed Al-0.13 wt.% Mg alloy, shear banding was found to be the main deformation mechanism [45]. The author of this publication indicates that orientation splitting involving fine scale irregular deformation banding is probably the most significant source of grain refinement and shear banding, with being dominant in the 2nd pass ECAP also providing an important mechanism for grain subdivision.

Farshidia et al. 2018 confirmed that shear bands occurred in Nb, Fe-20Cr, and TNTZ already in the first ECAP pass [46].

Studies using the CEC method have shown that in Al99.992 and Al99.5 aluminum and AlMg5 alloy, the primary deformation mechanism in the extreme strain range is shear in the shear bands and micro shear bands [47]. The work carried out clearly confirms previous results from CEC and other SPD methods.

The presented literature data prove that severe plastic deformation processes lead to structure refinement due to shear bands propagation.

**Figure 20:**
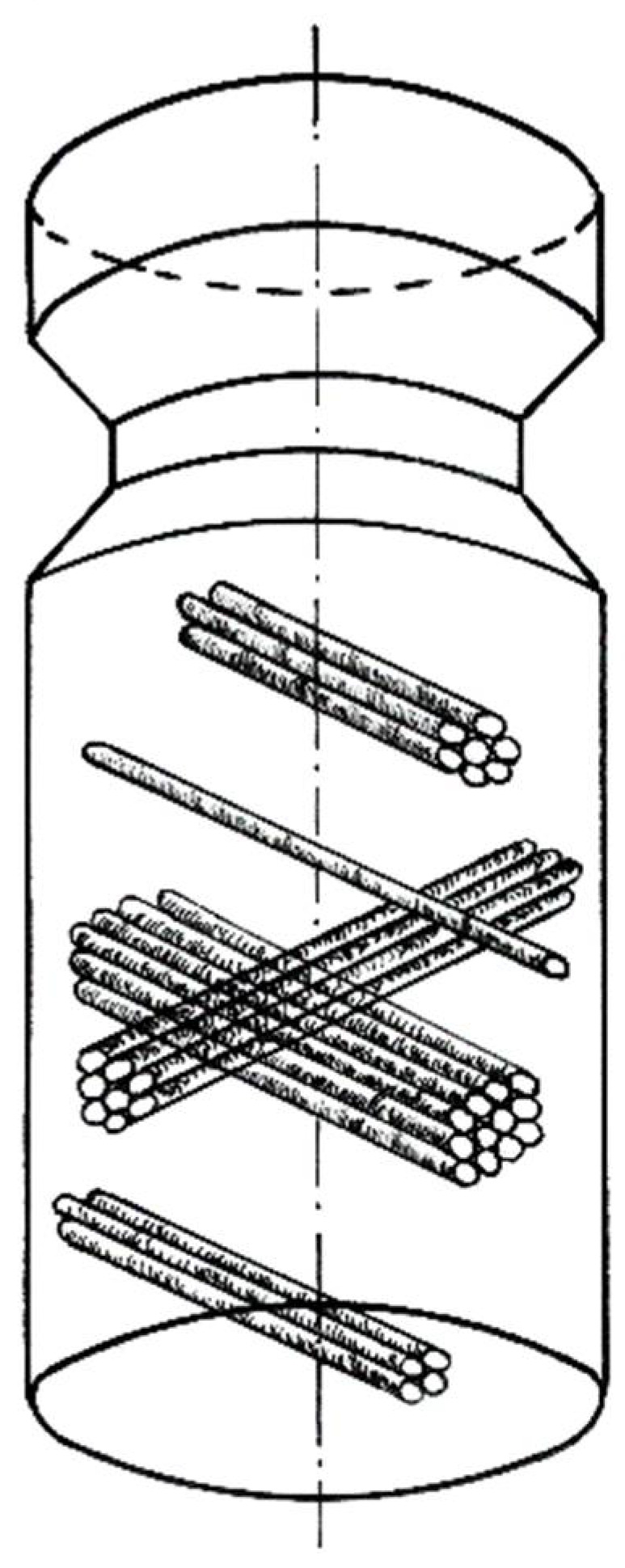
Scheme of crossing shear bands in CEC sample.

In shear bands, strain accumulates during deformation, which leads to the formation, in an initially homogeneous material, of zones of higher energy stored in defects of the structure located in the microbands and zones of lower stored energy in areas not yet deformed or subject to structure renewal processes such as recovery, polygonization or recrystallization. The highest dislocation density is found in the walls of the microbands. During reciprocating extrusion (CEC) deformation, the microbands intersect each other, which is due to the geometry of the process of extruding the sample through the narrowing of the CEC die (Figure 20).

The change in shape of the specimen forced by external forces occurs as a result of an increase in the intensity of slip in the shear bands, which successively fills the entire volume of the material (Figure 20). The occupation of the volume of the deformed metal by shear bands continues up to a certain strain value. This value of strain limit depends on the type of material, its stacking error energy, and the strain rate. If the deformation tools keep the specimen consistent and do not allow fracture to occur, mechanisms of structural softening are initiated so that further plastic deformation of the specimen can continue. The competitive interaction of the strengthening and renewal processes leads to a steady state flow. In this state, the equilibrium between the processes of hardening and the processes of structure renewal is established.

Figure 10, Figure 11 and Figure 12 illustrate the softening phenomenon in Al99.992 aluminum deformed by the CEC method, in which the high purity of the aluminum and the absence of prohibitive effects of admixtures, precipitates, or impurities blocking the grain boundaries resulted in the free development of dynamic recrystallization. The displacement of new grain boundaries was observed even after holding the sample at room temperature. Obviously, such a phenomenon would not be possible during conventional deformation due to too low accumulated strain energy.

CEC deformation, as in other SPD methods, produces a strongly differentiated microstructure. The resulting shear bands (Figure 13) are adjacent to strongly recovered, polygonised, or recrystallized areas. There are very soft (recrystallized or polygonised) and strongly hardened (shear bands) areas in the samples. This creates significant gradients of stored energy. The geometrically necessary dislocations (GNDs) and statistically stored dislocations (SSDs), and their impacts on the hardness variation during annealing treatments for highly deformed aluminum alloy were investigated by Jandaghi M.R. et al. [8]. Microstructure survey revealed that generated shear-bands by CGP acted as talent sites for further strain-induced grain boundary migration (SIGBM) during annealing.

A vector field assigns a vector quantity to each point in space. A scalar field is manifested by giving the value of the field at selected points or by connecting points of equal value with lines or surfaces. The gradient of the scalar field defines the vector field. It would be possible to draw isolines of equal material density (in areas of high dislocation density the material density should be lower) or equal value of accumulated energy (dislocation density could be a measure) and create maps showing variations in the level of stored energy indicating a potential tendency to change the microstructure. Estimation of gradients of stored energy in a given area could provide information on the expected tendency to form a granular or nanometric structure.

The size of nano and micrograins mainly depends on the geometrical parameters of the shear bands, most importantly on their width. This is due to the fact that the grain size is larger along the direction of the microbands. Within the boundaries of the microbands there are accumulated defects in the structure, impurities, or micro-precipitations, forming a specific region of high energy potential in relation to the poorly defected interior of the microbands. It is likely that in multiphase metals, or those containing alloying elements, such boundaries prevent the growth of nucleating grains.

The study of Luan et al. 2021 showed that in pure aluminum, nucleation of new grains occurs preferentially in strain bands and shear bands [48]. In CEC-deformed Al99.992 aluminum, new grains were also observed in shear bands. This phenomenon is macroscopically shown in Figure 7 and Figure 8. The enlarged fragment of the structure in Figure 7 and the new grains at the boundary in Figure 8 also indicate that the boundaries of old grains can be the site of nucleation of new grains. However, in the case of nucleation of new grains at the boundaries, it should be noted that they are located at the intersections of the boundaries with the bands. Figure 8b further indicates that the grain near the boundary after 12 days of holding at room temperature grew in the opposite direction to the original direction of new grain growth associated with the dynamic recrystallization process. This would imply that the driving force for the movement of the new grain boundary is due to the accumulated energy in the bands. Therefore, it is likely that the presence of shear bands rather than the presence of old grain boundaries is more important in the nucleation of new grains, which would confirm the study of Luan et al. [48].

The study of Al99.992 aluminum provides clear evidence for the initiation of nuclei in shear bands. Due to the development of microbands throughout the volume of the samples, it is possible to develop nanometric structures by SPD methods. These methods provide sufficient energy accumulation to enable nucleation of new nano and micro grains and their growth.

It can be assumed that in the range of deformations well above conventional strains, the accumulation of defects in the boundaries increases the misorientation angles. This is also confirmed by work carried out on 1050 aluminum [49] Cu–Al alloys [50]. The studies suggest that the higher the strain, one would potentially expect a larger volume of material with nanometric features with large misorientation angles. However, this relationship is hindered by structure renewal and softening processes, annihilating defects and contributing to the reduction in misorientation. The higher the strain, the annihilation of defects can proceed more intensively, especially in metals with higher stacking fault energy, which is probably the reason for the reduction of the average size of the misorientation angle in Al99.5 aluminum after 67 CEC cycles. Moreover, the nanometric structure can be produced only in a certain part of the sample volume.

In this study, steady-state flow was achieved for Al99.992 aluminum after only 2 deformation cycles φ = 1.8, for Al99.5 aluminum after 5 CEC deformation cycles φ= 4.5, and for AlMg5 after deformation of φ = 15 (36 CEC cycles). The lower the stacking fault energy, the more multiphase the material, the greater the strain required to reach the steady state. This results in a greater ability to produce nanometric, pseudo-nanometric, or granular structures in a larger volume of the material being deformed.

In light of the obtained results and literature data, SPD processes are difficult to be applied in metal technologies. The expected properties may not be achieved, particularly with respect to nanometric grain size refinement. Moreover, the large accumulation of strain energy may result in instability of the structure, as exemplified by aluminum Al99.992 deformed by CEC method.

Upon analysis of the phenomena involved in the generation of volumetric nanometric structure by SPD methods, significant barriers emerge that prevent the complete transformation of the original structure into a nanomaterial. Even the deformation of metals and alloys with low stacking fault energy containing particles, as well as multiphase metals, does not ensure the complete transformation of the structure into a nanometric structure due to the inevitable achievement of a steady-state flow that stabilizes the grain size and property level. In the steady state, as a result of the balance between the processes of hardening and renewal of the structure, depending on the material, a grain structure is formed, the size of which affects the level of hardening.

Despite structural phenomena hindering mass production of large volume bulk nanomaterials, the application of SPD technology has significant prospects. It is possible to refine the structure of aluminum and its alloys to nano- and micrometric sizes in the range of several tens of micrometers, while in conventional plastic processing the commonly obtained structure has grain sizes of about several hundred micrometers. Significantly reducing the grain size can result in higher strengthening of pure aluminum. On the other hand, the refinement of grains to nanometer size will promote plasticization of aluminum alloys.

## 5. Conclusions

The research presented and its analysis based on our own work indicates that:

1. Microbands and shear band formation is the primary deformation mechanism for Al99.992 and Al99.5 aluminum, AlMg5 alloy, and AlCu4Zr alloy in the CEC method.
2. Microbands fill the entire volume of CEC deformed specimens as strain increases, and their intersection is a source of microstructural change.
3. After an initial increase in hardness, which in Al99.992 aluminum occurs up to φ = 1.8 (2 CEC cycles), in A99.5 aluminum up to φ = 4.5 (5 CEC cycles), and in AlMg5 alloy up to φ = 15 (36 CEC cycles), a steady-state flow condition characterized by a constant value of strengthening was found.
4. The level of steady-state properties depended on the type of material being deformed and was the result of the competitive interaction of hardening and structural renewal processes such as recovery, polygonization, and recrystallization.
5. A granular microstructure with nanometer and micrometer sizes was observed in the steady-state flow range.
6. Dynamic and post-dynamic recrystallization with the formation of new grains in areas of shear bands was observed in Al99.992 aluminum deformed by CEC method.
